# Optimization of the Balanced Steady State Free Precession (bSSFP) Pulse Sequence for Magnetic Resonance Imaging of the Mouse Prostate at 3T

**DOI:** 10.1371/journal.pone.0018361

**Published:** 2011-04-08

**Authors:** Christiane L. Mallett, Paula J. Foster

**Affiliations:** 1 Department of Medical Biophysics, The University of Western Ontario, London, Ontario, Canada; 2 Imaging Research Laboratories, Robarts Research Institute, London, Ontario, Canada; Cornell University, United States of America

## Abstract

**Introduction:**

MRI can be used to non-invasively monitor tumour growth and response to treatment in mouse models of prostate cancer, particularly for longitudinal studies of orthotopically-implanted models. We have optimized the balanced steady-state free precession (bSSFP) pulse sequence for mouse prostate imaging.

**Methods:**

Phase cycling, excitations, flip angle and receiver bandwidth parameters were optimized for signal to noise ratio and contrast to noise ratio of the prostate. The optimized bSSFP sequence was compared to T1- and T2-weighted spin echo sequences.

**Results:**

SNR and CNR increased with flip angle. As bandwidth increased, SNR, CNR and artifacts such as chemical shift decreased. The final optimized sequence was 4 PC, 2 NEX, FA 50°, BW ±62.5 kHz and took 14–26 minutes with 200 µm isotropic resolution. The SNR efficiency of the bSSFP images was higher than for T1WSE and T2WSE. CNR was highest for T1WSE, followed closely by bSSFP, with the T2WSE having the lowest CNR. With the bSSFP images the whole body and organs of interest including renal, iliac, inguinal and popliteal lymph nodes were visible.

**Conclusion:**

We were able to obtain fast, high-resolution, high CNR images of the healthy mouse prostate with an optimized bSSFP sequence.

## Introduction

Prostate cancer is the most-diagnosed non-melanoma cancer in Canadian men and the third-leading cause of cancer death [1]. Mouse models of prostate cancer are valuable for pre-clinical studies of prostate cancer and include transgenic models such as the Transgenic Adenocarcinoma of Mouse Prostate (TRAMP) model [2], and xenograft models [3]–[5] that typically involve subcutaneous or orthotopic (intra-prostatic) injection of cancer cells. Subcutaneous tumours are easy to implant and measurable with calipers, but orthotopic tumours are superior for studies in which metastasis is desired [6]–[9]. In the case of intra-prostatatic tumours, without imaging, tumour volume can only be estimated by palpation and then measured after sacrifice, which requires a single measurement for each animal and potentially a large number of animals with separate groups of mice sacrificed at multiple timepoints.

The use of magnetic resonance imaging (MRI) allows for the non-invasive quantification of tumor size, so that multiple timepoints are measured in each animal, allowing for smaller sample sizes and more complete data. There is also the opportunity to manipulate the tissue contrast to better visualize the tissue of interest and to gain different information about pathology. The prostate is adjacent to the bladder and surrounded by fatty tissue, which must be taken into account when determining which imaging pulse sequence and parameters to use. MRI has been used to monitor prostate tumour growth in mice, primarily at high field strengths (>4T), but also at clinical field strengths (1.5T and 3T). Most investigations have used 2D T1- or T2-weighted spin echo sequences (T1wSE and T2wSE), but 3D imaging sequences have also been used [9]–[23].

The purpose of this study was to optimize 3D imaging of the mouse prostate to achieve high SNR, high CNR and high resolution between the prostate and surrounding tissues, using the balanced steady state free precession (bSSFP) pulse sequence. This SNR-efficient sequence has not previously been used for mouse prostate imaging, and has the advantage of sensitivity to iron, which will be useful in future studies of iron-labeled cell tracking in a mouse model of prostate cancer.

## Methods

### Animals

Healthy male nude mice (5–12 weeks of age) were studied. Mice were housed in a specific pathogen-free barrier facility in between scanning sessions. All animal experiments were approved by the Animal Use Subcommittee of the University Council on Animal Care at The University of Western Ontario following the guidelines of the Canadian Council on Animal Care (protocol # 2006-03).

### MRI

Scans were performed on a 3T GE Excite MR750 system using a custom-built high-performance gradient insert with an inner diameter of 17.5 cm, maximum gradient strength of 500 mT/m and peak slew rate of 3,000 T/m/s, and a custom solenoidal whole-mouse body RF coil 4 cm in length and 3 cm in diameter. For live mouse imaging, mice were anaesthetized with isoflurane (2% in oxygen) and placed supine in the coil, warm saline bags were taped near the RF coil to maintain body temperature, and the mice were wrapped with gauze and tape for consistent positioning and to minimize motion artifact due to respiration. For ex vivo imaging, a mouse was euthanized by euthanyl then immediately scanned in the same manner.

Images acquired using the bSSFP pulse sequence had the following parameters at 200 µm isotropic spatial resolution. For axial scans, the field of view (FOV) was 3×3 cm (14 minutes) or 4×4 cm (20 minutes), and for coronal scans it was 6×3.3 cm (26 minutes). The flip angle (FA) was varied between 30°, 40° and 50°. The receiver bandwidth (BW) was varied from ±31.25, ±41.67, ±62.5 and ±83.3 kHz. Repetition time (TR) was automatically set by the scanner software in accordance with BW and FOV, and echo time (TE) was set to be half of TR. Thus, TR ranged from 3.3–4.6 ms and TE from 1.7–2.3 ms. The number of signal averages (NEX) ranged from 1–4. An RF phase cycling scheme with a sum of squared reconstruction was implemented and the number of phase cycles (PC) was varied between 2–8. Axial bSSFP images (FOV 3×3 cm, 14 minutes) were compared with the more traditionally-used spin echo (SE) images acquired with the following parameters: axial orientation, FOV 3×3 cm, TR/TE = 600/25 ms (T1w), 2000/70 ms (T2w), 1 mm slice thickness, 128×128 matrix, 234 µm in-plane resolution, and acquisition time of 20 (T1w) or 17 (T2w) minutes.

### Image Analysis

Images were compared based on signal to noise ratio (SNR), contrast to noise ratio (CNR), and presence of artifacts such as chemical shift. SNR was calculated as the mean signal from the hindlimb muscle divided by the standard deviation of the background signal. CNR was calculated as the difference in SNR between the prostate and the surrounding fatty tissue. In order to compare sequences with different scan times and slice thicknesses, SNR efficiency was calculated as the SNR divided by the square root of the scan time (in minutes) and was normalized by slice thickness (in mm).

## Results

### Effect of phase cycles and averaging

**Figure 1:**
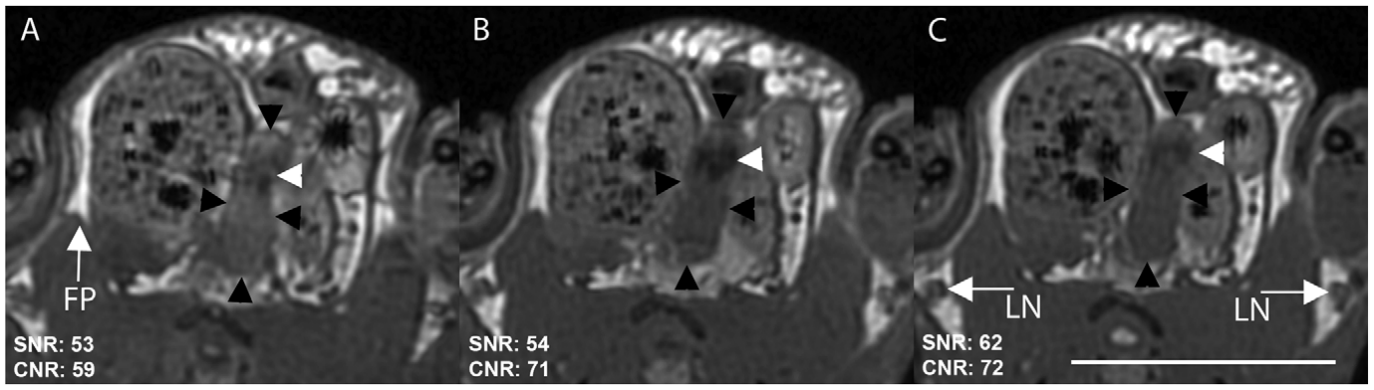
Effect of phase cycling and averaging on ex vivo prostate image quality. Cropped and enlarged sections of axial scans: A: 2 PC, 4 NEX, B: 4 PC, 2 NEX, C: 8 PC, 1 NEX. Black arrowheads indicate prostate, white arrowheads urethra, FP is the fat pad used for CNR measurements and LN are the inguinal lymph nodes. Scale bar is 1 cm. Axial scan, FOV 4×4 cm, 200 µm isotropic resolution, TR/TE = 3.9/2.0 ms, FA 30°, BW ±62.5 kHz, 20 minutes.

Phase cycling is used with the bSSFP sequence to avoid the appearance of characteristic dark banding artifacts that are caused by sensitivity to local field inhomogeneities and which degrade image quality considerably. Figure 1 shows the effect of phase cycling (2, 4 and 8 PC) and averaging (4, 2 and 1 NEX) on prostate image quality in a sacrificed mouse. All scans took 20 minutes. SNR values did not vary significantly with different amounts of phase cycling, and CNR was highest for 4 PC, 2 NEX and 8PC, 1 NEX. There was no banding artifact in any of the images. The shape of the prostate in these ex vivo images is different from the prostate in vivo due to deflation of the bladder in the sacrificed mouse. For all future bSSFP acquisitions, 4 PC and 2 NEX were used.

### Effect of bandwidth and flip angle

**Figure 2:**
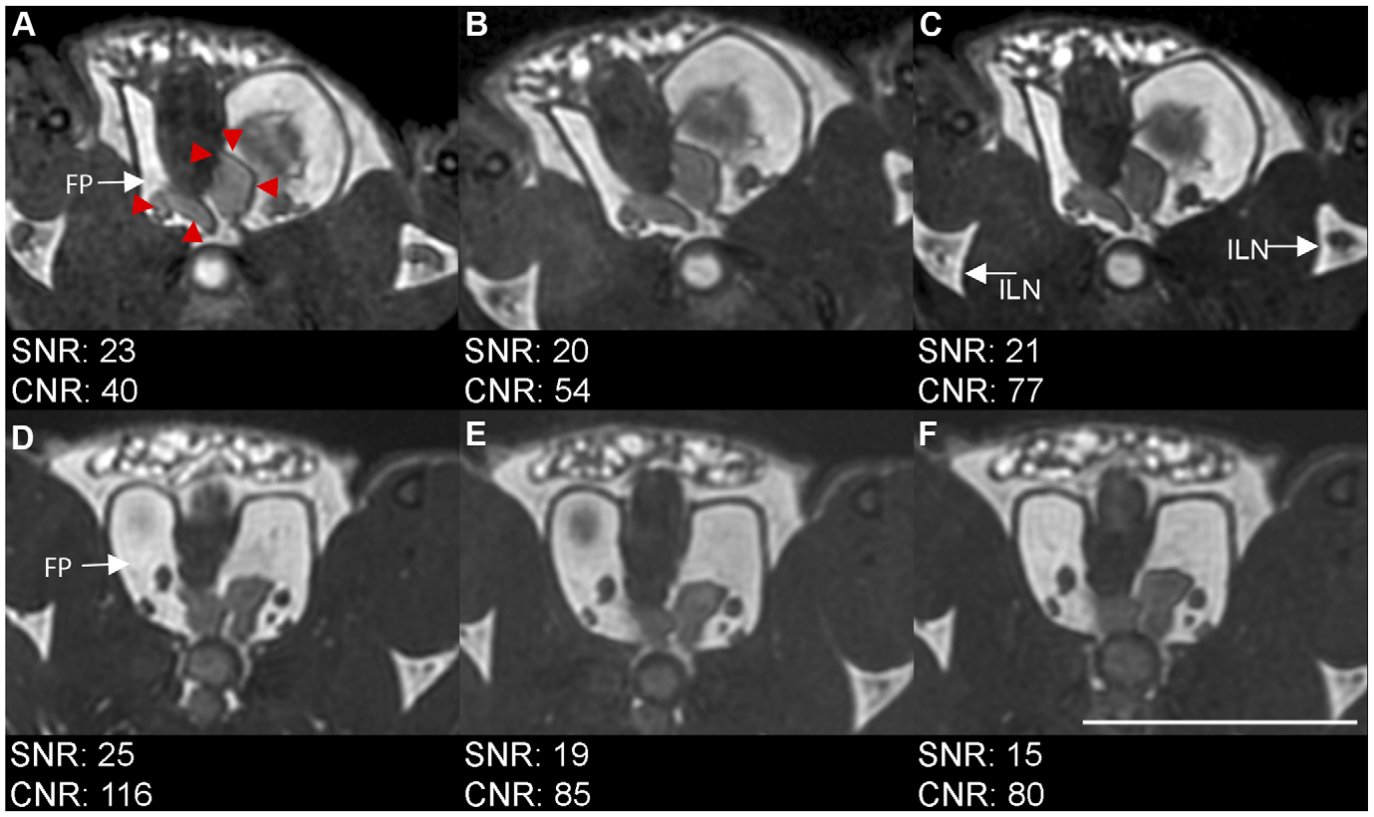
Effect of flip angle and bandwidth on prostate visibility and artifacts. Flip angle of A: 30° vs B: 40° vs C: 50° at BW of ±62.5 kHz. Bandwidth of D: ±31.25 kHz vs E: ±62.5 kHz vs F: ±83.3 kHz. Red arrowheads indicate prostate boundaries. White arrows point to fat pad used for CNR calculations (FP) and to inguial lymph nodes (ILN). Scale bar is 1 cm. Scan parameters: Axial scan, FOV 3×3 cm, 200 µm isotropic resolution, TR/TE = 3.3–4.6 ms/1.1–2.3 ms, 4 PC, 2 NEX, 14 minutes.

When flip angles were compared, image SNR (based on muscle signal) was approximately equal between flip angles, ranging from 20 to 23 (Figure 2). CNR increased with flip angle, with values of 40, 54 and 77 for 30°, 40° and 50°, respectively. With a flip angle of 50°, the best SNR and CNR was obtained with a bandwidth of ±31.25 kHz (SNR = 25, CNR = 116); however, there were artifacts such as a slight chemical shift between the urethra and prostate tissue, as well as a blurring of the edges of the prostate, at the lowest bandwidth (D) compared to the highest bandwidth (F). When the bandwidth was set to ±62.5 kHz, the artifacts were reduced with a higher CNR than was seen at a bandwidth of ±83.5 kHz.

### Comparison of bSSFP with T1w and T2w SE

**Figure 3:**
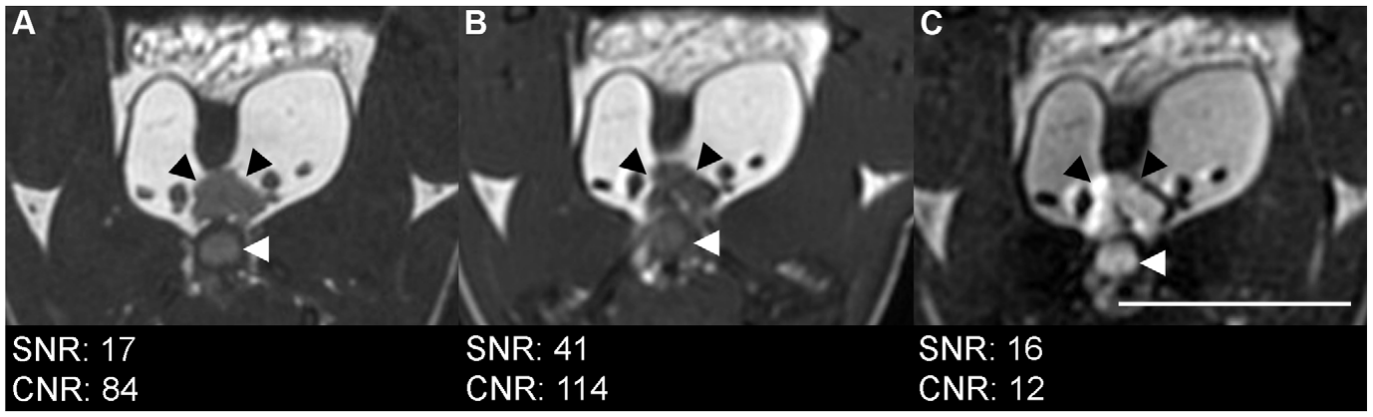
Comparison of in vivo axial views acquired with A: bSSFP, B: T1wSE and C: T2wSW. Black arrows indicate prostate, white arrows indicate urethra. Scale bar is 1 cm. bSSFP images acquired using optimized sequence with 3×3 cm FOV. Spin echo sequences acquired with axial orientation, FOV 3×3 cm, TR/TE = 600/25 ms (T1w), 2000/70 ms (T2w), 1 mm slice thickness, 128×128 matrix, 234 mm in-plane resolution, and 20 (T1w) and 17 (T2w) minutes acquisition time.

Axial scans of a mouse were acquired with bSSFP with 4 PC, 2 NEX, FA 50° and BW ±62.5 kHz (14 minutes) as determined above and compared to T1wSE (20 min) and T2wSE (17 min) scans with parameters as indicated in the methods section (Figure 3). The T1wSE image had the highest CNR of the prostate relative to the surrounding fat (114), compared to 84 for the bSSFP image and 12 for the T2wSE. While the overall SNR was highest for the T1wSE image at 41, it must be noted that the slice thickness for the bSSFP, which had an SNR of 17, was 0.2 mm, compared to 1 mm for T1wSE. The SNR for the T2wSE, also acquired with 1 mm slices, was 16. The SNR efficiency was calculated and normalized by slice thickness: the bSSFP had the largest SNR efficiency at 23, compared to 9 for the T1wSE and 3 for the T2wSE.

### 3D nature of bSSFP

**Figure 4:**
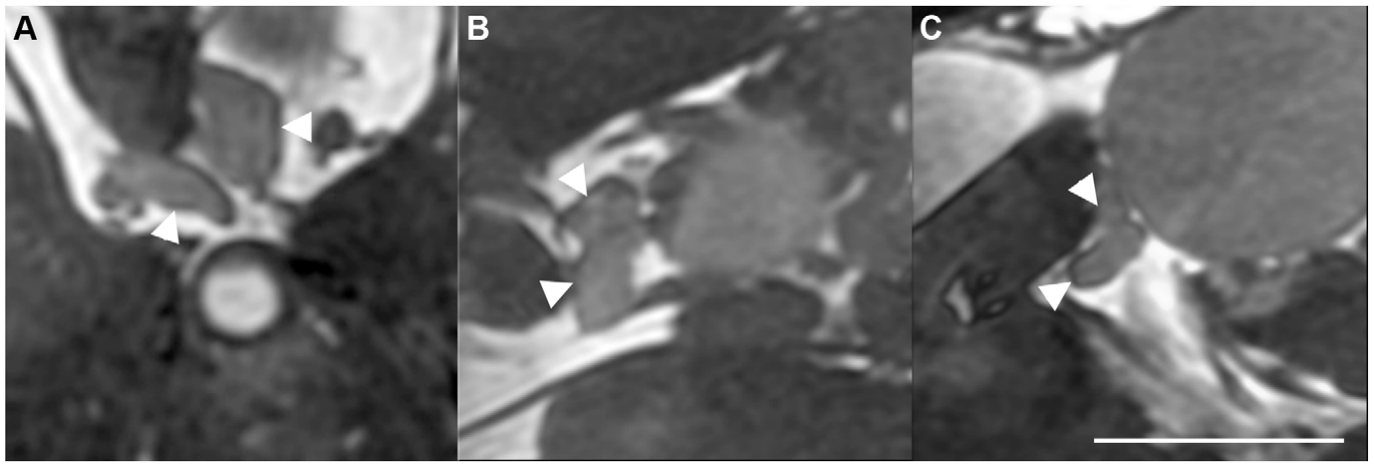
3 views of prostate from one in-vivo scan. A: axial, B: coronal, C: sagittal. White arrows indicate prostate. Scale bar is 0.5 cm. Axial scan, FOV 3×3 cm, 200 µm isotropic resolution, TR/TE = 4.6 ms/2.3 ms, 4 PC, 2 NEX, FA 50°, BW ±62.5 kHz, 14 minutes.

**Figure 5:**
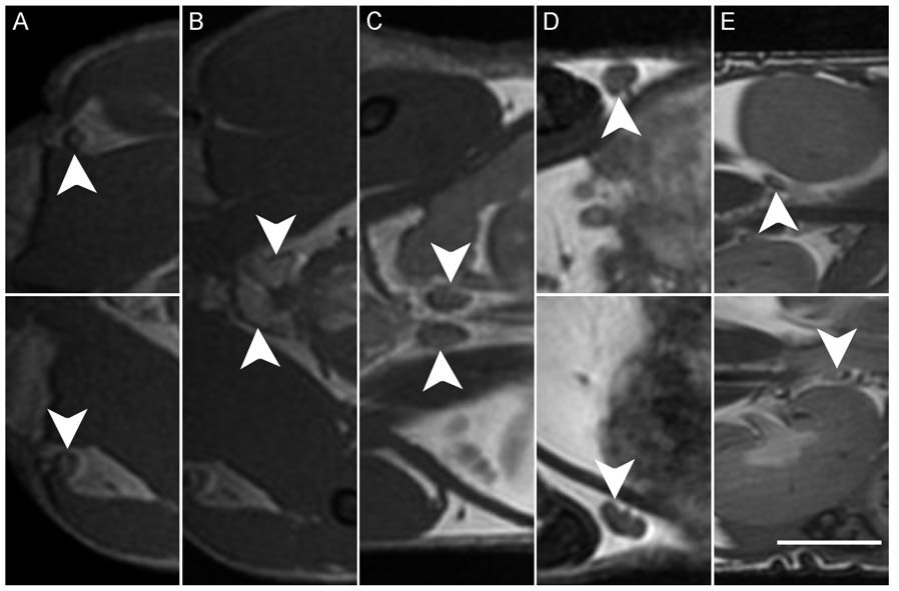
Sections of coronal view of mouse with prostate and lymph nodes identified. Tail is at left, head at right. White arrows indicate organs of interest as follows. A: popliteal lymph nodes; B: prostate; C: iliac lymph nodes; D: inguinal lymph nodes with lymph vessels visible; E: Renal lymph nodes. Scale bar is 0.5 cm. Coronal scan, FOV 6×3.3 cm, 200 µm isotropic resolution, TR/TE = 4.6/2.3 ms, BW ±62.5 kHz, FA 40°, 8 PC, 2 NEX, 26 minutes.

Since bSSFP is a 3-dimensional sequence, the image can be re-oriented to view the prostate from any angle (Figure 4). This is valuable to visualize the morphology and size of the prostate. A simple re-orientation of the scan plane and enlargement of the field of view allows for acquisition of whole mouse-body images, in a short scan time, that include clear views of the prostate and other organs of interest, such as lymph nodes and lymph vessels (Figure 5).

## Discussion

MRI measurements of the mouse prostate are desirable for visualizing the prostate over time with the flexibility of being able to manipulate contrast. An advantage of MRI is sensitivity for early detection: measurements of the long and short axes of the prostate in 2D T1wSE images acquired at 7T were able to detect prostate cancer 4 weeks sooner than by palpation [12], [16].

Previous work on imaging the mouse prostate has been primarily with 2-dimensional T1W [12], [16], [19], [21] or T2W [9], [11], [18]–[20], [22], [23] spin echo pulse sequences that provide only a single orientation for viewing. With these 2D sequences it is often necessary to use thick slices in order to obtain a reasonable SNR in a reasonable scan time – these have been as high as 500–2000 µm [14], [15], [19], [22], [23] sequences, although in one case it was reduced to 50 µm when field strength was increased to 4.7T from 1.5T [10]. In plane resolution is higher, typically ∼100 µm. Three-dimensional sequences such as T1W and T2W fast spin echo and fast low angle shot also yield a variety of slice thicknesses from 300–2000 µm and in-plane resolutions of ∼80–400 µm at clinical and high field strengths [11], [13]–[15], [17].

Even at high field strength, scan time can be quite long, for example 2.5 hours with an additional hour for setup [18]. While this protocol at 7T allowed for impressive discrimination of the ventral from the dorsolateral lobes of the prostate using a 2D T2WSE sequence with CHESS (chemical shift selective sequence), this scan time is impractical for studies involving more than a few mice. More reasonable scan times of 10–15 minutes at 7T were achieved through the use of techniques such as RARE and multi-echo sequences [9], [11].

Techniques for enhancing prostate contrast include using a long TE and fat saturation in a 5–15 minute 2D fast spin-echo (FSE) scans at 3T [19]. Also at 3T, a 3D fast low-angle shot sequence with fat suppression was used to obtain scans with 400 µm isotropic voxel size in 10 minutes [17]. Additionally, fat suppression by saturation pre-pulses has been used at 7T [9], [17]–[19]. Gadolinium has also been used to enhance contrast [21], [24]. Other methods of visualizing prostate tumours include using diffusion weighted imaging, which improved detection of small tumours (<1mm in diameter) compared to T2W imaging in a transgenic mouse model of prostate cancer [20].

In this study, we did not use any additional contrast enhancement techniques such as fat suppression; consequently, the seminal vesicles were not detectable from the surrounding fat in healthy mice. It is common for prostate tumours to spread to the seminal vesicles; however, the seminal vesicles can be completely destroyed by large prostate tumours [16], and the tumour-fat contrast may be different from healthy seminal vesicle-fat contrast. Nevertheless, it might be helpful in the future to exploit the chemical shift artifact of the second kind to suppress mixed water-fat pixels using a TE and TR such that the water and fat frequencies are out of phase; fat-tissue interfaces would be black. However, the TR would have to be increased, leading to a longer scan time [25].

The bSSFP pulse sequence is very SNR efficient and produces unique T2/T1 contrast [26]. This sequence has been recently applied to investigations of glioma in the mouse brain [27], [28]. A challenge presented by bSSFP, however, is its high sensitivity to local field inhomogeneities. The result is a characteristic “banding artifact” that worsens at higher field strengths and with longer TR [26]. Multiple acquisition RF phase cycling techniques ameliorate this problem and have allowed for bSSFP imaging at higher field strengths and with longer TR [28], [29].

Although the sensitivity of bSSFP to local field inhomogeneities can be problematic, it has also been what has enabled this sequence to be used for highly sensitive cellular imaging, which has allowed the detection of iron-labeled single cells and cell clusters at 1.5 T and 3 T [30]–[35]. This feature of bSSFP may be useful in mouse models of prostate cancer for detecting and monitoring metastases.

We have obtained excellent high resolution, high SNR images of the healthy mouse prostate in a relatively short scan time using the bSSFP pulse sequence. For our mouse studies, this was achieved using a custom-built high-performance gradient insert on a clinical 1.5T system. While the maximum strength of the insertable gradient coil used in this study is 500 mT/m, we operated below this. For example, with receiver BW of ±62 kHz, and FOV of 3cm, (gamma is 4257 Hz/g), the strength of the readout gradient is approximately 10g/cm = 100mT/m [G(readout) = 2*BW/(gamma*FOV)]. The gradient strength used to excite the slab is also far below this maximum strength since a thick slab is used that encompasses the whole mouse body. Clinical gradients of 50 mT/m and higher are now available on whole body scanners; therefore, b-SSFP protocols similar to that used in this study are not out of the question for modern-day clinical gradients.

In conclusion, this study shows that with optimized imaging parameters, 3D mouse body images acquired with bSSFP allow for the simultaneous visualization of the prostate and its draining lymph nodes, the iliac and renal lymph nodes, as well as the nearby inguinal and popliteal lymph nodes. The ability to detect both the prostate and the lymph nodes in a single fast, high-resolution scan will be useful for studies that aim to investigate prostate cancer metastasis.
